# Out of the shed and into the field: an immune toolkit for measuring wild ungulate immune phenotypes at multiple scales

**DOI:** 10.1093/discim/kyag001

**Published:** 2026-03-05

**Authors:** Luke Weinstein, Brian P Dolan, Holly K Arnold, Sara Carpenter, Leigh Combrink, Clinton W Epps, Jennifer L Johns, Emma Lantz, Brandon Munk, Shannon Phelps, Paige Prentice, Nicholas Shirkey, Marci Witczak, Anna E Jolles, Brianna R Beechler

**Affiliations:** Department of Biomedical Sciences, Carlson College of Veterinary Medicine, Oregon State University, Corvallis, OR, USA; Department of Biomedical Sciences, Carlson College of Veterinary Medicine, Oregon State University, Corvallis, OR, USA; Department of Biomedical Sciences, Carlson College of Veterinary Medicine, Oregon State University, Corvallis, OR, USA; Department of Integrative Biology, Oregon State University, Corvallis, OR, USA; Department of Biomedical Sciences, Carlson College of Veterinary Medicine, Oregon State University, Corvallis, OR, USA; Department of Fisheries, Wildlife, and Conservation Sciences, Oregon State University, Corvallis, OR, USA; Department of Biomedical Sciences, Carlson College of Veterinary Medicine, Oregon State University, Corvallis, OR, USA; Wildlife Health Laboratory, California Department of Fish and Wildlife, Rancho Cordova, CA, USA; Wildlife Health Laboratory, California Department of Fish and Wildlife, Rancho Cordova, CA, USA; Department of Biomedical Sciences, Carlson College of Veterinary Medicine, Oregon State University, Corvallis, OR, USA; Department of Fisheries, Wildlife, and Conservation Sciences, Oregon State University, Corvallis, OR, USA; California Department of Fish and Wildlife, Sacramento, CA, USA; Wildlife Health Laboratory, California Department of Fish and Wildlife, Rancho Cordova, CA, USA; Department of Biomedical Sciences, Carlson College of Veterinary Medicine, Oregon State University, Corvallis, OR, USA; Department of Biomedical Sciences, Carlson College of Veterinary Medicine, Oregon State University, Corvallis, OR, USA; Department of Integrative Biology, Oregon State University, Corvallis, OR, USA; Department of Biomedical Sciences, Carlson College of Veterinary Medicine, Oregon State University, Corvallis, OR, USA

**Keywords:** bighorn sheep, eco-immunology, immunology, diagnostics, phenotypes

## Abstract

**Introduction:**

Understanding factors that shape immune responses in wild animals is critical to predicting population resilience and long-term persistence. Immune function modifies the survival of individuals facing infectious disease, trauma, and environmental stressors, yet remains understudied. An individual’s immune response is shaped not only by current and historic pathogen exposures but is mediated by both individual (e.g. host genetics, metabolic plane, age, and sex) and population-level (e.g. population size, density, and connectivity) factors. Bighorn sheep (*Ovis canadensis*, bighorn) occupy populations of varying sizes, nested within larger metapopulations, creating a hierarchical structure. This organization provides a useful framework to understand how immune parameters vary across individual, population, and metapopulation levels. Unfortunately, measurement of immune parameters in ungulates is limited.

**Methods:**

To address this limitation, we measured 18 immunologic traits across 581 wild bighorn to evaluate this toolkit’s ability to detect immunological differences between individuals, populations, and metapopulations.

**Results:**

Most immunological phenotypes illustrated significant variation at the metapopulation level and individual level. Our assays revealed immune phenotypic variation consistent with two main axes of segregation—one that distinguished tradeoffs in bighorn innate versus adaptive immune responses, and another reflecting alternative inflammatory states, defined by distinct cytokine patterns. Bighorn age and sex also mediated immune response patterns.

**Conclusions:**

Our immunological toolkit sets the stage to further clarify landscape-level immunological variation in wild ungulate populations and provides a template for deploying integrative eco-immunological tools in any natural population to further understand wildlife health.

## Teaser

Here we demonstrate the utility of our field-tested “immune toolkit” in describing the immune phenotypic heterogeneity of bighorn sheep (Ovis Canadensis) across individuals and large spatial scales within California.

## Graphical Abstract

**Graphical Abstract:**
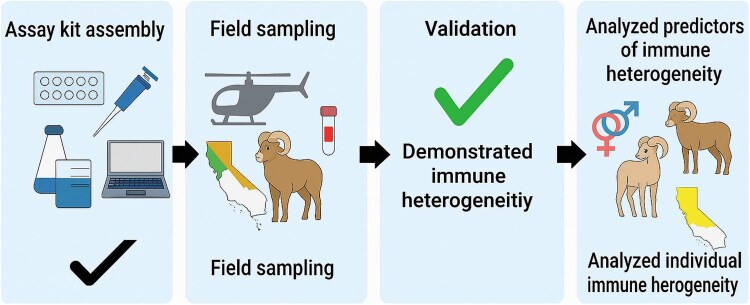

## Introduction

Determining the factors that drive the success of wild ungulates at many spatial scales is key to their long-term resilience. The importance of immunocompetence in the survival and success of populations is often underestimated. Infectious disease transmission and injury are among the primary drivers to elicit immune responses, which can be pivotal to an individual’s survival and the persistence of wild animal populations. Wild animals are typically exposed to a broad range of pathogens at any given point and must calibrate their responses to avoid immunopathology while also reducing pathogenic burden. Here, immunopathology is used to describe adverse consequences to the host from its own immune system.

Immune function in wild animals is affected by both current and historical exposure to pathogenic and parasitic agents, but the response to challenge is constrained by the traits, genetics, and metabolic status of the host. The co-evolution of parasites and the host immune response is well described in numerous systems, and the history of exposures may lead to the development of specific immunogenetic profiles [1] that constrain the immune response generated in an individual when exposed to a pathogen or parasite. Additionally, most wild animals live in variable environments where nutritional limitations are common—animals in negative energy balance make different investments in immune responses than those in positive energy balance [2]. Lastly, factors including age and sex are also key to understanding variance in wildlife immunocompetence [3–5].

Immunocompetence is a broad term that encompasses a variety of physiological responses in a host. The immune response is a complex combination of cellular and protein components that respond together in variable ways to pathogenic challenge. For instance, the initial response to a pathogen is primarily mediated by the innate immune response, which in turn determines the suite of adaptive responses that are mounted by the host. Therefore, when evaluating host immune responses, it is beneficial to consider an integral approach to studying the response, considering multiple measures across multiple levels of response. Studies that focus on whole-host immunity in concert rather than in terms of isolated responses can elucidate patterns of immune heterogeneity in wild animals better than attempting to use isolated measures of immunity [4, 6–8]. By understanding these patterns, researchers can then interpret and predict patterns of immune phenotypic variation.

Delineating immune functions into specific categories can help researchers understand complex interactions as the host utilizes multiple types of responses in concert during immune activation due to pathogen exposure or trauma. Here, we define specific suites of responses as immune phenotypes-expressed in the host and detected in our assay data. Broadly, immune responses can be defined as involving innate or adaptive immunity. Innate immunity is primarily responsible for the acute response to trauma or infection in the form of damage-associated molecular patterns (DAMPS) or indications of nonself cells in the form of pathogen-associated molecular patterns (PAMPS). This type of immunity responds to broad categories of damage or infection and is less precisely tailored to the specific pathogen at hand [9]). Adaptive immunity produces cells and signals in response to innate immune activation that resist future infections by the same or a similar pathogen. This type of immunity is generally elicited to abrogate the effects of specific pathogens during and after infection. When effectively deployed in tandem, these responses are essential for the continued health of wild animals.

When host immune systems are stimulated, an inflammatory response is often elicited. In mammalian immune systems, this includes a suite of responses associated with innate and acquired immunity to clear pathogens at the source of invasion, prevent further infection, and begin the process of cellular repair. Cytokines, small protein immunomodulators, are released during inflammation and guide the immune response. As a result, these small protein immunomodulators can be used to indicate the severity and category of inflammation the host is undergoing. Tumor necrosis factor alpha (TNFα) and interleukin (IL)-10 are two cytokines classically associated with pro- and anti-inflammatory signaling, respectively. TNFα is released in response to proinflammatory stimuli, including other cytokines, as well as injury or pathogen detection by the host. IL-10 is often released to modulate the potential for host cell damage that may arise from inflammatory signaling. IL-4 is associated with classic T helper 2 (Th2)-driven immune responses. Th2-driven immune responses are less acutely pro-inflammatory than their Th1 counterparts [10]. Separately, IL-10 is expressed by cells, including T regulatory cells (Tregs), and is involved with modulating immune responses by suppressing pro-inflammatory signaling. IL-6 is released in response to acute pathogen detection or indicators of trauma, and is not associated with longer-term immune activation [11]. IL-17a is an important cytokine released in part by Th17 cells and is associated with clearing microbial infections as a proinflammatory immunomodulator [12, 13]. Interferon gamma (IFNγ) is also a major proinflammatory cytokine that enhances cell-mediated antimicrobial immunity [14]. The levels of each of these cytokines circulating in the blood can determine the strength and type of inflammatory response the host mounts, and indicate the same for researchers. Thus, they can provide a picture of the potential cost of mounting an acute immune response and the consequences of sustained and severe states of inflammation in terms of tissue damage [11, 15, 16]. A severe acute phase inflammatory response may clear infection from a pathogen quickly, but may also cause host damage as a casualty, while being energy intensive. Conversely, a more moderate response may limit the damage to the host yet modulate clearance of the pathogen [17, 18].

When measuring a host immune response, investigators can choose to evaluate constitutive or stimulated components. Constitutive immunity here refers to baseline immune activity that occurs without experimental stimulation and provides a lens into the current immune status of the animal upon sampling. In contrast, stimulated responses can be conducted *in vivo*, or more commonly, on cultured cells *in vitro*, to understand how host immune cells are able to react to potential or actual pathogenic threats. Taken in combination, a more complete host immunophenotype can be investigated—revealing not only a host's baseline immunological status, but its ability to respond to pathogen infection at the time of sampling.

**Figure 1:**
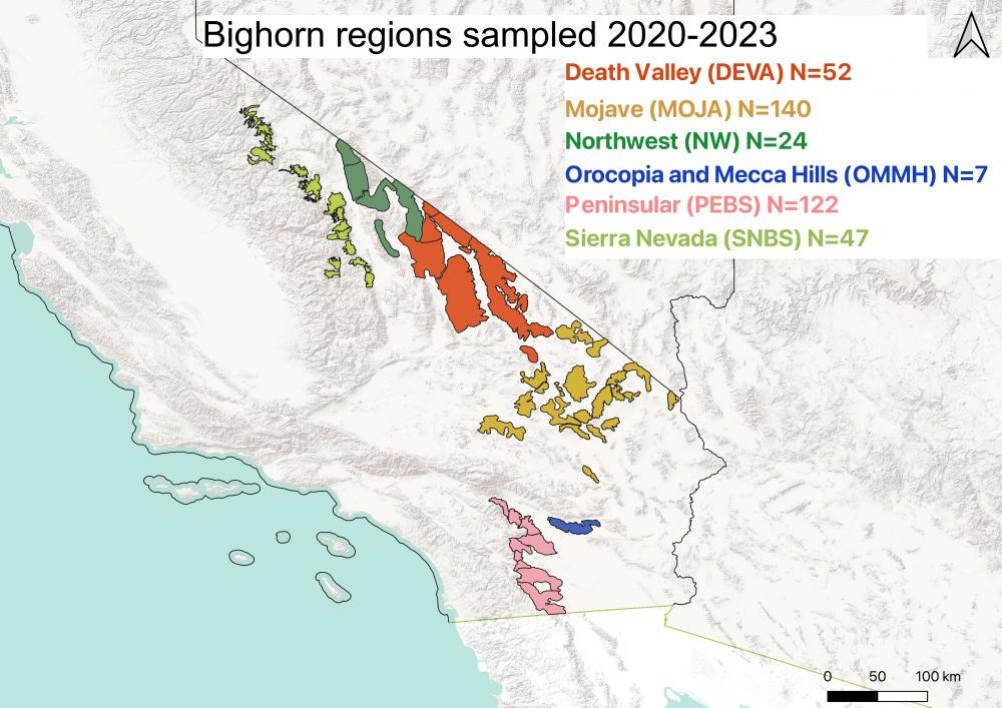
Map of bighorn capture sites from 2020 to 2023. Color corresponds to the relative region, and the numbers are representative of those bighorn used in our data analysis for this work.

In our study, we assembled an immunological toolkit to evaluate immune phenotype with the aims of characterizing inflammatory activation, understanding innate/adaptive tradeoffs, and evaluating both stimulated and constitutive responses. We chose to demonstrate this toolkit in a study system of bighorn, which includes two subspecies: desert bighorn sheep (*Ovis canadensis nelsoni,* bighorn) and Sierra Nevada bighorn sheep *(Ovis canadensis sierrae,* bighorn*),* spread across a wide and varied geographic area of California, USA (Fig. 1). Bighorn in this system occupy relatively discrete, arid mountain ranges as population units within well-described metapopulations and regions [19–22] representing distinct evolutionary trajectories with differences in genetic heterozygosity, population isolation, habitat quality, predation pressures, and pathogen presence [23, 24]. These systems of populations allowed us to ask about spatial variation in immune phenotype, as each region is relatively discrete from one another genetically and in terms of pathogen transfer. Here we divide our bighorn into regions that represent relatively isolated groups of populations that are expected to have such variation and have, if any, limited pathogenic or genetic exchange with other regions. Moreover, bighorn are long-lived hosts with phenotypic sexual dimorphism, allowing us to evaluate the role of sex and age in individual immune phenotypes. We expected age and sex to be significant drivers of individual immune phenotypic differences, as at the time of capture, most adult bighorn males and females are undergoing different physiological and behavioral changes in response to the reproductive season (rut) and pregnancy, respectively. Because bighorn are long-lived hosts, we expected to see differences in adult bighorn immune phenotypes due to variation in accrued pathogenic exposures, habitat, and predation-related behavior and trauma. This phenotypic variation is expected to also demonstrate the variety of strategies for activating or modulating the immune response in a wild animal that is subject to myriad pathogenic challenges, among other threats, including predation. Here, we seek to explore the use of a panel of immunological assays (Table 1) selected to assess bighorn sheep immune phenotype and ask whether this panel of assays can demonstrate immunological heterogeneity across age, sex, and geographic structure.

**Table 1:** Immune measures of bighorn investigated, divided into constitutive and stimulated responses.

Parameter	Outcome measured	Difficulty in field	Assay success	Median	IQR
Constitutive Responses					
Neutrophils (%) Differential WBC Count	Percent of white blood cells that are neutrophils	1	95%	44	34–53
Lymphocytes (%) Differential WBC Count	Percent of white blood cells that are lymphocytes	1	95%	43	34–52
Monocytes (%) Differential WBC Count	Percent of white blood cells that are monocytes	1	95%	3	2–5
Eosinophils (%) Differential WBC Count	Percent of white blood cells that are eosinophils	1	95%	8	4–13
Basophils (%) Differential WBC Count	Percent of white blood cells that are basophils	1	95%	0	0
IFNγ [pg/ml] Plasma concentration	Amount (pg/ml) of IFNγ in plasma	2	98%	0.4	0.2–0.8l
IL-4 [pg/ml] Plasma concentration	Amount (pg/ml) of IL4 in plasma	2	98%	43.3	20.9–110.6
IL-6 [pg/ml] Plasma concentration	Amount (pg/ml) of IL6 in plasma	2	98%	8.5	1.4–28.2
IL-10 [pg/ml] Plasma concentration	Amount (pg/ml) of IL10 in plasma	2	98%	123.1	58.4–245.7
IL-17a [pg/ml] Plasma concentration	Amount (pg/ml) of IL17 in plasma	2	98%	1.2	0.2–4.8
TNFα [pg/ml] Plasma concentration	Amount (pg/ml) of TNFα in plasma	2	98%	848.9	289.4–2296
Albumin [mg/dl] Plasma concentration	Amount (mg/dl) of albumin in plasma	2	98%	4.2	3.8–4.2
Globulin [mg/dl] Plasma concentration	Amount (mg/dl) of globulins in plasma	2	98%	4.3	3–4.3
Stimulated Responses					
Whole Blood Bacterial Killing Assay (WBBKA) [Percent killed]	Percent of bacteria killed by blood	3	95%	6.20%	0–16.8%
Plasma Bacterial Killing Assay Time to 50% growth (PBKA 50%) [Percent killed]	Proportional difference in hours in how fast the bacteria grow to 50% when plasma is present compared to when no plasma is present	2	98%	0.1%	0–0.25%
Plasma Bacterial Killing Assay Max Killed (PBKA Max) [Percent killed]	Percent of bacteria killed by plasma	2	98%	6.60%	0–25.8%
Lymphocyte Proliferation Assay Colorimetric Detection LPS stimulated (LPA LPS) [Proportion activated]	Proportional difference between lymphocyte activity in wells with ConA compared to wells without	3	86%	−0.11	−0.1–0.1
Lymphocyte Proliferation Assay Colorimetric Detection ConA stimulated (LPA ConA) [Proportion activated]	Proportional difference between lymphocyte activity in wells with LPS compared to wells without	3	85%	−0.13	−0.29–0.02
PBMC IFNγ secretion assay [Proportional difference]	Proportional difference in IFNγ production by peripheral blood mononuclear cells stimulated with LPS compared to ones not stimulated	3	71%*	−0.12	−0.4–0.67

Difficulty of performing the assays in the field is ranked from 1 to 3, with 1 requiring minimal equipment, 2 requiring the use of a centrifuge and ability to freeze samples, and 3 requiring the use of continuous power and equipment. The number of times we successfully ran the assay out of 581 attempts (except IFNγstim, which had 484 attempts), as well as the median and interquartile range, is shown in the final 3 columns.

## Methods and materials

### Animal capture, release, and sample collection

Bighorn sheep were captured via helicopter net-gunning and were released in the autumns of 2020–2023 under appropriate federal and institutional approvals. National Park Service Permits for Scientific Research and Collecting were obtained from the Mojave National Preserve and Death Valley National Park (MOJA-2020-SCI-0031, MOJA-2023-SCI-0059, DEVA-2020-SCI-004). Additional National Park Service Institutional Animal and Use Committee–approved authorizations from the California Department of Fish and Wildlife (ACUP #PWR DEVA JOTR MOJA Epps Galloway Desert BHS 2019 A3, #CA MOJA Beechler Epps DBHS 2020 A2, and #CA DEVA Epps BHS 2022 A3). Oregon State University Institutional Animal Care and Use Committee (IACUC) approvals for all animal handling procedures were secured under protocols IACUC-2019-0017 (2019–2022), and IACUC-2022-0274 (2022–2025). All procedures and events described were in accordance with these permits, to ensure ethical treatment of animals, and compliance with regulatory standards. Animals were captured using a helicopter net-gun across southern California (Fig. 2.1, Table 2.1) and were released within an hour after capture. Animal processing typically after transport to base camp via helicopter, but when the weather was not permitting, processing occurred directly in the field. Animal characteristics, including sex, and age, were recorded. Using the emergence of incisors, and the number of horn rings, animals were classified into the age groups of young (0–<2 years), young adult (2–<6 years), and adult (6+ years). Blood samples were drawn from the jugular vein into sodium heparin, ethylenediaminetetraacetic acid (EDTA), and noncoated vacuum tubes, and then were transferred to a mobile laboratory, placed on ice, and processed within 8 hours of collection.

**Figure 2:**
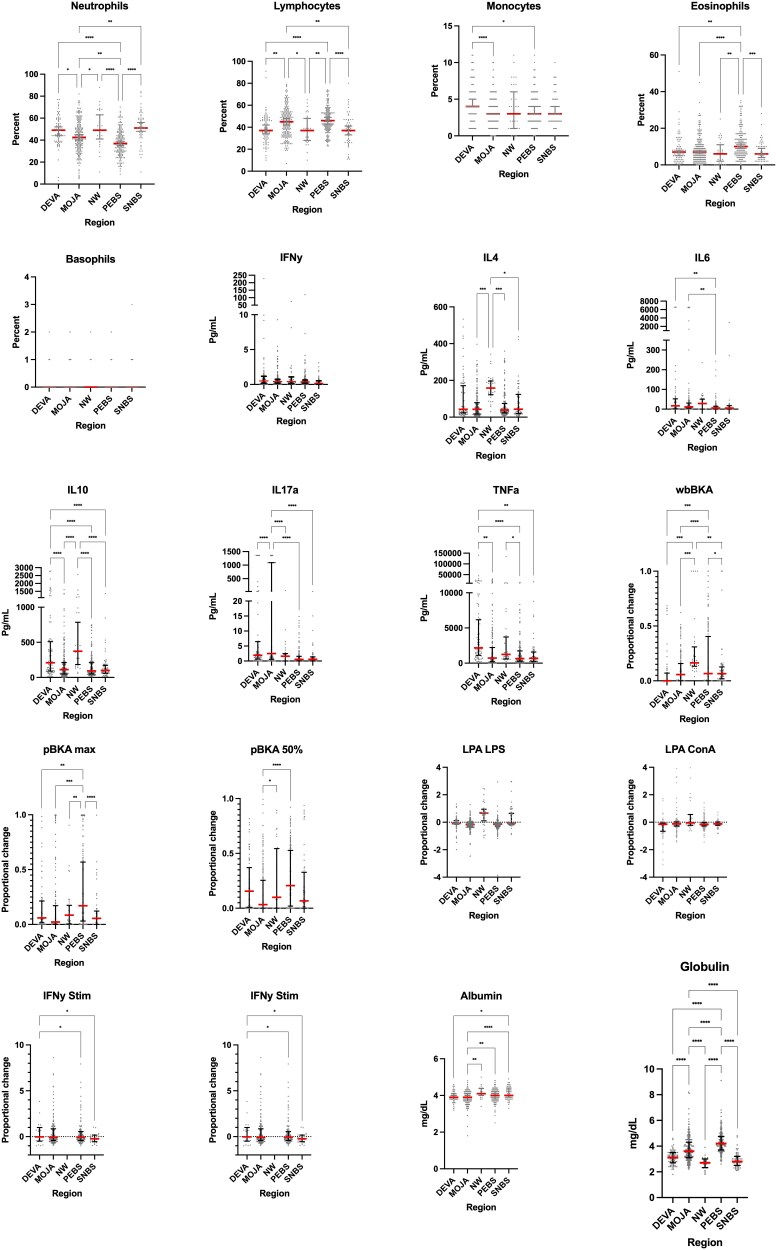
Distribution of each immune parameter for each region. The median is represented as a red line, the IQR is represented in error bars and each dot is a data point. Asterisks indicate a significant relationship between regions (One-way ANOVA, significant *P*-value ≤ 0.05). A * indicates ≤ 0.05, ** indicates ≤ 0.01, *** indicates ≤ 0.001 Regions are abbreviated as Death Valley (DEVA), Mojave (MOJA), Northwest (NW), Peninsular (PEBS), and Sierra Nevada (SNBS). Sample sizes are shown in Table 1 above. Note for IFNγ, IL6, IL10, IL17a, TNFα the y axis is split to better represent the bimodal distribution.

**Table 2:** Sample sizes and descriptive statistics by region, sex, and age class.

Region (sample size)	Age	Sample size	Sex	Sample size
DEVA (n = 52)	young	2	female	33
	2–5	30	male	19
	6±	20		
MOJA (n = 140)	young	9	female	91
	2–5	67	male	49
	6±	64		
NW (n = 24)	young	1	female	15
	2–5	11	male	9
	6±	12		
OMMH (n = 7)	young	1	female	4
	2–5	3	male	3
	6±	3		
PEBS (n = 122)	young	3	female	108
	2–5	46	male	14
	6±	73		
SNBS (n = 47)	young	9	female	31
	2–5	21	male	15
	6±	17		

Samples were processed at the point of capture using a solar-powered laboratory inside a van, allowing for live cell and laboratory assays. The van also contained liquid nitrogen tanks or coolers with dry ice for storage of samples below −80°C. The remaining assays (differential counts, cytokine assessment, plasma BKA, immunoglobulin, albumin) were performed on frozen stored blood products, which only required the use of a centrifuge and dry ice or liquid nitrogen in the field—which was also contained within our mobile laboratory, but could be performed with a simpler laboratory setup (generator, truck, and centrifuge).

### Hematology

Blood collected in serum vacutainer tubes without anticoagulant was allowed to clot before being centrifuged at 3000 times gravity (RCF) for 10 minutes, and the serum supernatant was then transferred to a new tube. Fresh whole blood from heparinized green top vacutainer tubes was centrifuged at a minimum of 3000 RCF for ten minutes, and the plasma supernatant was transferred to a microcentrifuge tube. Serum and plasma samples were stored on dry ice in the field and then transferred to a −80°C freezer upon return to the laboratory.

Two blood smears per sample were prepared in the field using blood from a potassium EDTA (purple top) vacutainer tube and were stored in a dry container until fixation. Slides were fixed within ∼1 month of collection and stained with Hematik stain pack using a Siemens Hematic stainer. Blood smear slides were processed routinely by the clinical pathologists at Carlson College of Veterinary Medicine to manually determine differential white blood cell (WBC) counts, counting 100 leukocytes and recording the relative proportion of lymphocytes, neutrophils, monocytes, eosinophils, and basophils.

### Cytokine analysis

We assessed the presence of cytokine concentration in plasma using a Milliplex magnetic bead kit (Millipore) read on an LX-200 instrument. Cytokine concentrations were determined using a custom 6-plex ovine cytokine assay developed by Merck-Millipore (SCTY1-91K, Millliplex xMAP, Merck-Millipore, France). The analytes included two cytokines involved in the innate immune response (IL-6, TNFα) and four cytokines involved in the adaptive immune response (IL-4, IL-10, IL-17A, IFN-γ). Assays were carried out on a set of 96-well plates over the course of weeks, either as a single plate or in batches of two plates. The majority of plates were run by a single researcher, with the rest conducted under this person’s supervision. Each plate included its own high and low concentration control as well as a plate-specific set of standards from which the curve was derived. Using GraphPad Prism (GraphPad Software, La Jolla, CA), we determined net median fluorescence by extrapolating third-order polynomial measurements from a standard curve to determine the raw amount of pg/ml of cytokines in each sample. These samples were compared between individuals as well as within individuals as a ratio (e.g.. TNF-α:IL-10).

### Blood biochemistry

Serum blood biochemistries were derived routinely by the Oregon Veterinary Diagnostic Laboratory (OVDL) [25]. Values included in our immunological toolkit included albumin and immunoglobulins. Stored serum was submitted to Oregon State University Veterinary Diagnostic Laboratory (Corvallis, OR) for blood chemistry analysis. These included values of albumin (g/dl) and immunoglobulin (g/dl).

### Whole Blood Bacterial Killing Assay

Fresh whole blood from a heparinized green top vacutainer tube was used within 8 hours of sample collection for the Whole Blood Bacterial Killing Assay (WBBKA) described previously [26, 27]. In brief, a pellet of lab-strain *Escherichia coli* (Epower microbiologics, ATCC 8739) was resuspended in 37°C phosphate-buffered saline (PBS) to a concentration of 10^5^ bacteria/ml. Approximately 200 ul of whole blood was vortexed and diluted with complete media into concentrations of 1:800 and 1:1600. Next, 40 µl of complete media was pipetted into a 96-well round-bottom plate. All experiments were performed in duplicate. Forty microliters of whole blood dilutions were added to respective experimental wells, while 40 µl of additional complete media was added to each control well. All wells then received 10 µl of the resuspended *E. coli* solution. To minimize edge effects, the assay was performed exclusively in interior wells, while exterior wells were filled with 40 µl of PBS to avoid plate edge effects. Plates were shaken gently to mix contents and then incubated at 37°C for 1 hour. After incubation, 125 µl of prewarmed Tryptic Soy Broth (TSB) was added to all experimental wells, and then gently shaken. Baseline absorbance was measured using a spectrophotometer (300 and 600 nm wavelength), and then plates were incubated for 12 hours at 37°C. Assay absorbance at 12 hours was measured as before. To analyze the readings, the reading at time zero was subtracted from the same wells’ reading at 12 hours for control and sample wells, respectively. This is considered the sample and control difference values (Time 12 − time 0 = average difference). Duplicates were averaged and then revised to zero out any negative values to establish both the average control difference and the filtered sample difference, respectively. To determine the proportion of bacteria killed, the average control difference was divided by the filtered sample difference (for each duplicate sample), and the resulting value was then divided by the average control difference (Equation [1]):


(1) $$\mathrm{Proportion}\;\mathrm{bacteria}\;\mathrm{killed}=\frac{\left(\mathrm{average}\;\mathrm{control}\;\mathrm{difference}-\mathrm{filtered}\;\mathrm{sample}\;\mathrm{difference}\right)}{\mathrm{average}\;\mathrm{control}\;\mathrm{difference}}.$$

### Plasma Bacterial Killing Assay

Plasma stored as described was used in the plasma bacterial killing assay previously delineated [26, 27]. In brief, under sterile conditions, a pellet of lab-strain *E. coli* (Epower microbiologics, ATCC 8739) was resuspended in 37°C PBS to a concentration of 10^5^ bacteria/ml. Approximately 100 µl of plasma was diluted with PBS into concentrations of 1:20 and 1:40. 40 µl of PBS was added to each control well, and 40 µl of each solution per sheep in duplicate was added to each corresponding well. Ten microliters of the bacterial solution was then added to all wells prior to incubation for 30 minutes at 37°C. After incubation, 125 µl of previously prepared sterile TSB was added to each well and then gently shaken. The opacity was measured using a spectrophotometer (300 and 600 nm wavelength), and then the plates were incubated at 37°C. Using this method, this strain of bacteria is known to begin its exponential growth phase after 4 hours [27]. At 4 hours and every subsequent hour thereafter, the absorbance was measured by spectrophotometry at 300 and 600 nm wavelengths and plotted as a function of time to establish a growth curve. This curve was fitted to a sigmoidal growth curve using GraphPad Prism™ (GraphPad Software, La Jolla, CA) to establish the time at which 50% killing was established. To establish the overall maximum killing, the starting absorbance at each wavelength was subtracted from the final absorbance—as described in the calculation for whole blood BKA (Equation [1)].

### Peripheral blood mononuclear cell isolation

Fresh whole blood in sodium or lithium heparin tubes was transferred within 8 hours of collection to Lymphoprep (Progen) tubes previously filled with a specific-gradient solution and centrifuged at 1000 RCF according to the manufacturer’s instructions to isolate the peripheral blood mononuclear cell (PBMC) layer. If Lymphoprep tubes were not available, we used Ficoll centrifuged at 1000 RCF to achieve the desired separation of layers (we determined no differences in the methodologies here other than a higher yield from the lymphoprep tubes). The PBMC layer was extracted via pipettor and deposited into a 15-ml conical tube containing 5 ml of PBS for further centrifugation at 1000 RCF for 5 minutes. The presence of a cell pellet was verified prior to decantation of the supernatant and resuspension of the pellet in 500 µl of PBS via vortexing. Cell concentration was measured by mixing a 10% solution of cells in Trypan blue and counted on a hemocytometer using a phase contrast microscope.

### Lymphocyte proliferation assay colorimetric detection

Prior to cell isolation, lipopolysaccharides (LPS, *E. coli*-derived) and Concanavalin A (ConA) were diluted to solutions of 0.00001 and 0.001 mg/ml, respectively. Following isolation of PBMCs as described earlier, PBMC cells were diluted to 10⁶ cells/ml in Aim-V complete media. A volume of 100 µl each of the cell dilution (10⁵ cells) was pipetted into 8 wells of a 96-well round-bottomed plate in duplicate for a total of 16 experimental wells per animal. If the plate had empty wells, 100 µl of PBS was added to wells around the experimental wells to reduce plate edge effects. For each set of 16, eight wells were given 100 µl of Aim-V complete media as controls, four wells were given 100 µl of the LPS working solution, and four wells were given 100 µl of the ConA working solution prior to incubation for 48 hours at 37°C. After 48 hours, 40 µl of Alamar blue was pipetted into two ConA and two Control wells per individual prior to incubation of the plate for a further 24 hours. The absorbance at wavelengths 500 and 600 nm was recorded by a spectrophotometer at 72 hours, while 20 µl of alamar blue was then pipetted into two of the LPS and two more control wells prior to further incubation. The absorbance at wavelengths of 500 and 600 nm was recorded again by a spectrophotometer at 96 hours of incubation. The resulting value of lymphocyte proliferation was determined by the following equation: [(Experimental average − control average)/control average] (Equation [2]):


(2) $$\mathrm{Lymphocyte}\;\mathrm{proliferation}=\frac{\left(\mathrm{experimental}\;\mathrm{average}-\mathrm{control}\;\mathrm{average}\right)}{\mathrm{control}\;\mathrm{average}}.$$

### PBMC IFNγ secretion assay (tissue cytokine analysis)

During the LPA described earlier, the supernatant from two control wells and two LPS wells was pipetted into two previously labeled cryotubes and kept on liquid nitrogen or dry ice until storage at −80°C in the laboratory. These were then submitted to a commercial laboratory for IFNγ detection in the supernatant (RayBio Ovine Immunoquantitative IFN-gamma ELISA, product number IQO-IFNg). The proportion stimulated was then calculated as [(experimental well-control well)/control well] (Equation [3]).


(3) $$\mathrm{IFN}\gamma \;\mathrm{stimulated}=\frac{\left(\mathrm{experimental}\;\mathrm{well}-\mathrm{control}\;\mathrm{well}\right)}{\mathrm{control}\;\mathrm{well}}.$$

### Data analysis

We determined that our datasets were primarily not normally distributed in statistical terms (Shapiro–Wilk test, *P*-value <0.05 as significant) [28]. Animals present in the tails of our data distribution are included as representative of the spectrum of responses seen in a wild host. These animals with extreme values in some measures represent biologically relevant variation that helps explain spatial and individual-level differences in immune phenotypes.

Medians, interquartile ranges, and generalized linear models (GLMs) were calculated using the full set of available data for each assay; however, GLM’s excluded the Orocopia/Mecca Hills (OMMH) region due to sample size. However, for the correlation plot and ordination techniques, we omitted animals without complete immune data using the na. omit function in Rstudio [28]. Before using GLMs or ordinations, the immune measures were transformed by centering and scaling the data to account for differences in normality and scale of raw measures. All statistical tests were performed using custom scripts in R implemented within RStudio (version 2023. 2.1 + 402). To test if immune measures were significantly explained by animal population, age, and sex, we modelled each immune measure as a function of age, sex, and region (glm: Immune_Measure ∼ Age + Sex + Region). GLM outputs were further compared in a pairwise fashion using the emmeans function to compare categorical outcomes [29]. All pglm results are publicly available on Figshare [30]; https://doi.org/10.6084/m9.figshare.30680816). To explore meaningful associations of assay results at the individual level, we created correlation matrices [31]. To determine the distribution of our data, we generated violin plots [32]. To visualize the Euclidean distance between immune measures at the individual level, we created a principal components analysis (PCA) and displayed the primary drivers of that statistical space in a bar chart [33–35]. For the PCA, we defined strong positive and negative correlation coefficients as >0.4 and <−0.4, respectively, while we defined moderate correlation coefficients as >0.2 and <−0.2, respectively. To assess sexual, age class, and regional differences in immune phenotypes in Canberra space, we performed a principal coordinates analysis (PCoA) and ran an Analysis of Variance (ANOVA) test of the centroid differences between categorical variables [34]. To assess beta-dispersion of the PCoA output, we performed a Tukey-based ANOVA.

## Results

### Field-testing an eco-immunological toolkit for wild bighorn

We evaluated immune responses in 581 wild bighorn from six geographic areas in California (Table 1). Eighty-three individuals were captured in the Death Valley (DEVA) metapopulation, 35 in the Northwest Death Valley (NW) region, 207 in the Mojave (MOJA) metapopulation, 11 in the OMMH region, 182 in the Peninsular metapopulation (PEBS), and 63 in the Sierra Nevada region (SNBS), respectively (Fig. 1). We tested a trial panel of immune assays including constitutive (WBC counts, albumin and immunoglobulin, and a plasma cytokine panel) and stimulated (lymphocyte proliferation assays, bacterial killing activity of whole blood and plasma, and IFNγ secretion by PBMCs to mitogenic stimulation) immune assays (Table 1). To evaluate the utility of these assays for quantifying immune variation in wild bighorn, we considered the practicality of running each assay under field conditions (Table 1), and the ability of the test to recover meaningful immunological variation among individual bighorn and across regions (Fig. 2). When isolating PBMCs from whole blood there was little difference in data quality between ficoll separation of PBMCs and the use of Lymphoprep tubes other than in time saved and a slightly increased yield of undiluted cells.

We found that our mobile lab allowed for successful performance for most of our assays in producing data that accurately and precisely described bighorn immunological phenotypes and the heterogeneity amongst the animals. The only assays that did not produce usable data reliably were the two lymphocyte proliferation assays, which required 72 hours of incubation in the field (Table 1 and Fig. 2). The performance of these tests was inconsistent in terms of capturing the biological variation among individual bighorn and bighorn regions, making the resulting data less informative. Neither of the colorimetric lymphocyte proliferation assays we ran showed significant variation with animal sex, age, or among regions; however, the IFNγ secretion by mitogenic stimulation assay did reveal some variation by individual and region (Fig. 2). Plasma cytokine levels consistently detected differences among individual bighorn and geographic regions, but not among individual male and female sheep, or animals of different ages. By contrast, leukocyte differential counts, blood biochemistry parameters (albumin and immunoglobulin), and bacterial killing assays performed well at capturing the biological variation both among bighorn regions and among individuals of different demographic groups.

### Defining immune phenotypes in bighorn

We used PCA and pairwise correlations among immune measures to evaluate immune phenotypic patterns in our study population of bighorn.

**Figure 3:**
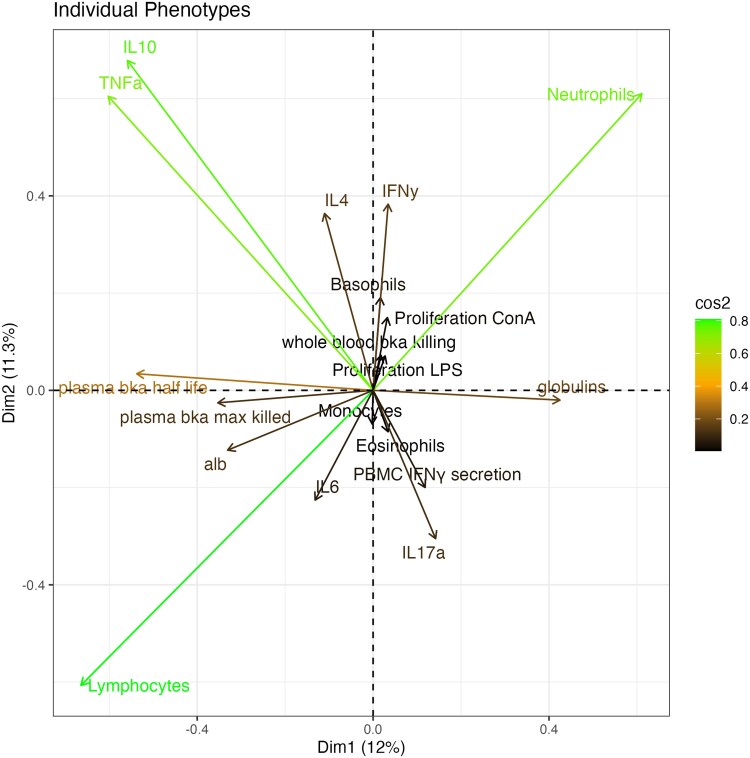
Immunological variation among bighorn. A: PCA demonstrating the axes of bighorn immune responses. Larger and greener vectors indicate greater contribution to the dimensional space N = 392.).

Our PCA suggests that bighorn immunological patterns segregate primarily according to two axes, defined by contrasts in (1) the relative abundance of lymphocytes as well as eosinophils versus neutrophils in the animals’ leukocyte counts, and (2) alternative inflammatory states marked by high levels of TNFα and IL-10 versus an elevation in Ig, IL-6, and IL-17a (Fig. 3). For simplicity, we subsequently refer to these axes as an ‘adaptive—innate’ axis and ‘inflammatory phenotype’ axes. Pairwise correlations among immune measures within individual bighorn support these findings. We identified strong negative correlations between neutrophils versus lymphocytes and eosinophils, positive associations between TNFα, IL-10, IL4, and IFNγ, and a positive association between IL-6 and IL-17a. In addition, our measures of bacterial killing ability (pBKAmax, pBKA half-life, wBKA) were positively correlated, as one might expect (Fig. 3, Supplementary Tables S1 and S2).

Examination of the distribution of data for each immune measure revealed that the six cytokines (IL-10, TNFα, IFN**γ**, IL-4, IL-6, IL-17a) defining the inflammatory phenotype axis exhibit bimodal distributions. In our sample of bighorn, most animals had cytokine levels close to the sample median, but for each of these cytokines, a subset of animals had strikingly elevated levels illustrative of immune activation in some cases by 1–3 orders of magnitude (Fig. 2 and Supplementary Figure S1). Together, our analyses suggest that these individuals were either marked by elevated IL-10, TNFα, IL4, and IFN**γ**, or composed a second group of outlier animals that had secreted high levels of IL-6 and IL-17a. As such, the inflammatory phenotype axis was driven by two groups of animals with distinct patterns of cytokine elevation.

Interestingly, we did not detect any negative associations between the cytokines marking the two different inflammatory phenotypes. As such, these appear to be two independent inflammatory response patterns composed of contrasting syndromes of cytokine activation, perhaps representing responses to different infectious challenges or groups of bighorn with distinct immunogenetic backgrounds.

### Demographic and geographic drivers of immune phenotypic variation in bighorn

**Figure 4:**
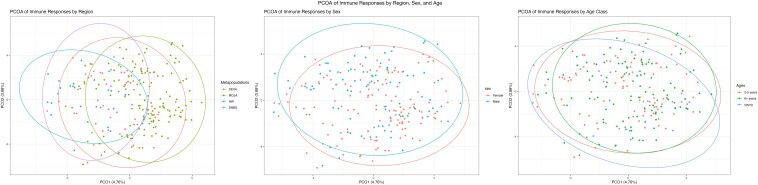
PCoA results of immune phenotypes by age class (a), sex (b), and region (c). using a Canberra distance matrix. N = 385 (RStudio version 2023.12.1 + 402, packages: vegan, corrr, and dyplyr). For age class (a), ellipses were significantly different by ANOVA of centroids (*P*-value 0.003). Beta dispersion was not significantly different between each age class as assessed by an ANOVA. For sex (b), ellipses were significantly different by ANOVA of centroids (*P*-value 0.001). Beta dispersion was not significantly different between each sex as assessed by an ANOVA. For region (c), beta dispersion was significantly different between each region as assessed by an ANOVA (*P*-value <0.05).

Immune phenotypic variation was, in part, driven by distinctive immune response patterns in bighorn at individual and regional scales. Young animals were relatively under-represented in our sampling (*N*_young_ = 25); nonetheless, young bighorn stood out as having high lymphocyte counts, lower neutrophil counts, stronger plasma bactericidal activity, and lower immunoglobulins compared to adults. Prime-aged adults (2–5 years) showed lower basophils, weaker blood bactericidal activity, lower immunoglobulins, and higher albumin than older (6+ years) adults (Supplementary Tables S1 and S2). These age-related immune-response patterns resulted in significant differences in overall immunophenotype by age group (Fig. 4).

When evaluating each of our component assays with respect to differences between males and females, only plasma bactericidal activity was stronger in female than in male Bighorn (Supplementary Tables S1 and S2).

Of the five regions we included in our sample population, bighorn in the MOJA, DEVA, and NW regions displayed striking immunological variation when compared to other regions. In almost every assay that we included (Fig. 4, Supplementary Table S2), we detected distinct regional immune phenotypes for these bighorn (*P*-value = 2.474 e−16; Fig. 4).

WBC populations varied across regions along a south–north axis, with the proportion of lymphocytes higher in Peninsular and Mojave bighorn than in Death Valley and Northwest populations, and lowest in Sierra sheep (Supplementary Tables S1 and S2, Fig. 2). Eosinophils and monocytes were also highest in Peninsular bighorn, while the proportion of neutrophils was highest in opposite regions to those with higher lymphocyte counts as one would expect. Bacterial killing by complement (plasma BKA max) followed geographic patterns of high lymphocyte differentials and low neutrophil differentials, perhaps reflecting alternative strategies of neutrophil- and complement -mediated killing of bacteria in blood. Accordingly, killing of bacteria by whole blood, which combines cellular and protein killing mechanisms, followed neither the neutrophil nor the pBKA geographic pattern.

Interestingly, variation in inflammatory phenotype may in part be driven by immune response patterns across regions. IL-10, TNFα, IFNγ, and IL-4, which defined one pole of our inflammatory phenotype axis above, were all elevated in Death Valley and NW populations, compared to Mojave, Peninsular, and Sierra Nevada populations. By contrast, IL-6 and IL-17a, markers of the opposite pole on our inflammatory phenotype axis, trended high in Mojave, Death Valley, and NW sheep, compared to lower levels among Peninsular and Sierra bighorn, again suggesting alternative inflammatory patterns, but no tradeoff between these phenotypes (Supplementary Tables S1 and S2, Fig. 4).

## Discussion

We curated a novel toolkit to study immunological variation in wild bighorn, an iconic species native to western North America, threatened by habitat fragmentation [35], climate change, and shifting disease exposures [26, 36, 37]. Our toolkit quantified 18 immune variables, including WBC differential counts, plasma-derived levels of six cytokines, bacterial killing by plasma and blood, lymphocyte proliferation, antibody titers, and albumin levels. We evaluated immune responses in 581 wild bighorn to test the practicality of our immune toolkit under field conditions and its utility in uncovering immunological variation among bighorn across populations in southern California.

### Field-testing an eco-immunological toolkit for wild bighorn

Our toolkit included assays with different levels of technical difficulty to perform in the field (Table 1). We were able to complete most assays in our lab van with a high success rate, except LPAs requiring long incubation times, which were hard to maintain during very cold outdoor temperatures and travel on unpaved roads. These inconsistent results have been reported previously in domestic sheep studies [38]. Cytokine release during mitogenic stimulation of PBMCs tended to yield usable data more reliably than colorimetric assays, because cytokine production tends to vary more across individuals than the number of metabolically active PBMCs, as measured in the colorimetric version of these assays [21]. Moreover, supernatant from the cell culture could be frozen and stored for later analysis in the lab using more sensitive equipment than the colorimetric LPA, which was performed entirely in our mobile lab in the field. As such, our study suggests that cell culture-based assays are feasible under field conditions in a mobile lab. However, it may be preferable to choose assays with a shorter incubation period (24 hours or less) and to quantify cell products using highly sensitive detection methods and specialized equipment back at a brick-and-mortar lab.

Assays that generate information on stimulated immune responses inform on the animal’s ability to respond to pathogen challenges; in contrast, constitutive immune measures provide a picture of the current state of the animal’s immunologic activation, which could be indicative of its recent exposures as well as its ability to mount immune responses. Because they confound exposure and response, constitutive immune measures can be more challenging to interpret than stimulated immune assays. Therefore, the added technical difficulty of conducting stimulated assays in the field may be justified, depending on the study goals.

Interestingly, we found that assays differed in the scale of variation that they were able to detect. All our assays (with the exception of the LPA, see earlier) revealed immunological variation among bighorn regions. Indeed, our analyses of immune phenotypes suggest that inclusion of WBC patterns and a cytokine panel reflecting a broad range of immune functions was essential to mapping immunological variation across populations. However, only a subset of assays—WBC differential counts and bacterial killing assays—was sensitive in a statistically significant way to immune response differences among individual bighorn. As such, immune toolkits for the study of wild bighorn and other ungulates might be optimized, depending on the questions at hand. Similar work, comparing the outcomes of immune assays to create toolkits representing a variety of immune responses have been curated in other wild species like mice [39]. Sometimes these toolkits are applied to test specific immune responses, like antibodies in Soay sheep [40]. Our findings thus expand a growing understanding of the utility of different assays in the study of immune response variation in natural mammalian populations.

### Defining immune phenotypes in bighorn

We used PCA and pairwise correlations among immune measures to understand immune phenotypic patterns in our study populations of bighorn. These analyses uncovered two dominant axes along which bighorn immune phenotypes segregated, corroborated by immune trait correlations concordant with these axes.

Bighorn immune phenotypes were defined by variation in emphasis on adaptive versus innate immune responses, represented in our PCA by an axis driven by high lymphocyte and eosinophil counts, versus high neutrophil counts; and the fractions of lymphocytes and neutrophils were strongly negatively correlated. This correlation is unsurprising, since neutrophils and lymphocytes are the most common cell types among WBCs of mammals [41–43]; Tizard, 2018), including bighorn [44]. However, there was no *a priori* expectation that this tradeoff defines a dominant axis of immune phenotypic variation among bighorn. Demographic and geographic drivers both appear to play a role in mediating the balance between adaptive and innate-linked WBC fractions in our study population of bighorn (see later). Younger bighorn and from more southern geographic areas (e.g. Mojave, Peninsular ranges) tended to have relatively higher lymphocyte fractions.

The second axis in our PCA of immune phenotypes segregated bighorn by their inflammatory phenotype, as defined by co-elevated IL-10 and TNFα (which also correlated positively with IFN**γ** and IL-4), versus elevated IL-6 and IL-17a. The pattern of co-elevated IL-10 and TNFα levels is interesting because these cytokines typically oppose each other in function, with TNFα involved in upregulation of inflammatory immune responses [45], and IL-10 acting to downregulate inflammation, limiting its potentially harmful effects [46]. Increased expression of TNFα can be associated with chronic inflammation [47, 48]. In domestic sheep, IL-10 has been reported to have a larger effect on cell proliferation than cytokine production in domestic sheep [38]. As such, one possible explanation of the observed pattern of co-elevation is that bighorn in the Death Valley and NW populations, where this pattern predominantly occurred, may be experiencing chronic inflammation, perhaps due to an as yet unidentified pathogenic challenge, frequent injuries, or other chronic stressor. The timing of our study coincided with the bighorn reproductive season, when injuries—especially in males—are common, which could lead to elevated levels of inflammatory markers. However, we did not see a difference between male and female bighorn in the prevalence of TNFa/IL-10 co-elevation. While we are thus not certain about the underlying cause of this immune activation, it is plausible that the animals may be releasing IL-10 to modulate the adverse effects of unresolved inflammation, marked by prolonged stimulation of TNFα (and IFN**γ**).

The alternative inflammatory phenotype we observed was marked by elevated levels of the cytokines IL-6 and IL-17a, and was most associated with the Mojave, Death Valley, and NW regions, as opposed to Peninsular and Sierra metapopulations, which are genetically distinct from these central desert bighorn clades [22]. Both IL-6 and IL-17a are pro-inflammatory cytokines; IL-6 also plays a role as a myokine, released by muscles during exercise to mobilize extracellular substrates [49]. In its role as a pro-inflammatory cytokine, IL-6 is secreted by macrophages in response to pathogen-associated molecular patterns and mediates the acute phase response, including fever [50]. IL-17 plays a role in defenses against extracellular bacterial and fungal infections [13], especially of the respiratory tract [12], and also acts to regulate gut microbiota [51]. Why some bighorn displayed strongly elevated IL-6 and IL-17a levels or elevated TNFα, IL-10, and IFNγ will require deeper investigation, ideally including information on host immunogenetic background, exposure history, and current infections, along with physiological and environmental conditions.

### Bighorn immunity varies with differences in demography and geography

Our study demonstrated that bighorn experience marked changes in immune response patterns with age: their dominant WBC population shifts from lymphocytes to neutrophils; they accrue antibodies (globulins) over time, and along with these changes, bacterial killing by protein components declines with age while killing by cellular components (likely dominated by neutrophils) improves with age.

Similar shifts in WBC dominance have been observed in other ungulate species [52–54], and increases in immunoglobulin titer are consistent with previous observations in bighorn [44], perhaps reflecting a lifetime of pathogenic challenges and maturation of the immune system as documented in other ungulates. Changes in the relative efficiency of bacterial killing by whole blood and plasma suggest a shifting tradeoff between protein and cellular immune effectors [55, 56]. Neutrophils play a central role in blood bactericidal activity, whereas plasma bactericidal activity is mediated primarily by complement protein (Tizard, 2018). As such, the age-related increase in whole blood BKA may be related to the increase in the neutrophil fraction among WBCs.

We did not observe many differences in immune responses among male and female bighorn. This was surprising, given the well-documented effects of testosterone on immune responses [57–59], and the timing of our captures during the rut, when males experience high testosterone levels, tends to be in worse body condition, and suffer frequent injuries in the course of competition over females [60, 61]. When evaluating each of our component assays with respect to differences between males and females, only plasma bactericidal activity was stronger in females than males (Table 2.3). Nonetheless, our holistic PCoA analysis from our immune toolkit revealed significant differences between immune response patterns among male and female bighorn. These patterns were not revealed by models (glms) of individual immune responses, demonstrating the value of integrative immune phenotype assessment over comparing single assays across relevant groups.

The geographical region of origin was the strongest predictor in our study of immune phenotypic variation in bighorn. Our study covered a large geographic area in southern California, comprising vastly different habitats from high alpine biomes in the Sierra Nevada to low hot desert ranges across Death Valley, the Mojave Preserve, and Peninsular ranges. Many aspects of environmental variation may contribute to the differences in immune phenotypes that we observed. Forage quality and availability vary across the ranges bighorn inhabit due to differences in their elevation, rainfall, and temperature profiles [22]. These, in turn, affect the timing of life history events such as reproduction and parturition, as well as host nutritional status, all of which modulate immune allocation [2, 18]. Moreover, parasite and pathogen exposures of bighorn vary geographically [37, 62], as do microbiome communities in bighorn [63]—both of which are likely to affect immune response patterns [63]. Finally, bighorn across our study area display striking genetic population structure [20, 23], including immunogenetic variation that is likely to affect immune phenotypes [21, 26].

Immune patterns in natural host populations represent integrated responses to current and past immune challenges, constrained by the animals’ immunogenetic background and resources available to allocate to immune responses [64, 65]. As such, single immune response measures can be difficult to interpret because their meaning depends on the ecological and immunological context experienced by the hosts [4, 64]. The immunological toolkit we developed exemplifies an integrative approach to studying immune function in a wild mammal and sets the stage for future studies of bighorn immune function and its environmental, genetic, and physiological drivers. Our hope is that immune phenotypic data might complement long-term monitoring of California bighorn to clarify the role of disease in bighorn ecology and conservation.

## Supplementary Material

kyag001_Supplementary_Data

## Data Availability

The authors confirm that the data supporting the findings of this study are available within the article and its Supplementary materials. Additional data are available on request from the corresponding author, LAW. The data are not publicly available due to ongoing research with this data.

## Abbreviations

Bighorn: bighorn sheep, *Ovis canadensis*
DAMPS: damage-associated molecular patterns
PAMPS: pathogen-associated molecular patterns
TNFα: tumor necrosis factor—alpha
IL-10: interleukin 10
IL-4: interleukin 4
T_h_: T-helper cell
IL-6: interleukin 6
IL-17a: interleukin 17a
IFNγ: interferon gamma
WBBKA: Whole Blood Bacterial Killing Assay
PBKA 50%: Plasma Bacterial Killing Assay Time to 50% growth
PBKA Max: Plasma Bacterial Killing Assay Max Killed
LPA LPS: Lymphocyte Proliferation Assay Colorimetric Detection LPS stimulated
LPA ConA: Lymphocyte Proliferation Assay Colorimetric Detection ConA stimulated
RCF: relative centrifugal force
PBS: phosphate-buffered saline
TSB: Tryptic Soy Broth
WBC: white blood cell
PBMC: peripheral blood mononuclear cells
LPS: lipopolysaccharides
ConA: Concanavalin A
PCA: principal components analysis
PCoA: principal coordinates analysis
ANOVA: analysis of variance
DEVA: Death Valley Metapopulation
NW: North–West Death Valley Region
MOJA: Mojave metapopulation
OMMH: Orocopia/Mecca Hills region
PEBS: Peninsular metapopulation
SNBS: Sierra Nevada
OVDL: Oregon Veterinary Diagnostic Laboratory
GLM: generalized linear model
EDTA: ethylenediaminetetraacetic acid
