# Supplementary material for: Out of the shed and into the field: an immune toolkit for measuring wild ungulate immune phenotypes at multiple scales
Source: Discov Immunol. 2026 Mar 5;5(1):kyag001. doi: 10.1093/discim/kyag001 (PMC12961423; doi:10.1093/discim/kyag001)
Supplement: kyag001_Supplementary_Data [file kyag001_supplementary_data.zip › Supplement.docx]

**Supplement:**

Table S1: Column loadings of each immune parameter for the PCA. Table of each immune parameter’s contribution to the first two dimensions of the PCA. N=392.

| Variable | Dimension 1 percent loading | Dimension 2 percent loading |
| --- | --- | --- |
| Lymphocytes | 19.51 | 17.3 |
| Neutrophils | 16.51 | 17.5 |
| TNFa | 16.03 | 17.18 |
| IL10 | 13.8 | 21.63 |
| plasma bka 50% killing | 12.78 | 0.05 |
| globulins | 7.98 | 0.02 |
| plasma bka max killing | 5.51 | 0.03 |
| alb | 4.84 | 0.71 |
| IL17a | 0.89 | 4.37 |
| IL6 | 0.77 | 2.4 |
| PBMC IFNy secretion | 0.62 | 1.88 |
| IL4 | 0.54 | 6.22 |
| Eosinophils | 0.05 | 0.34 |
| Proliferation_ConA | 0.05 | 1.05 |
| IFNy | 0.05 | 6.89 |
| Proliferation LPS | 0.03 | 0.24 |
| Basophils | 0.01 | 1.71 |
| whole blood bka killing | 0.01 | 0.24 |
| Monocytes | 0 | 0.23 |

Table S2: Qualitative summary of generalized linear model results. The model used the form: glm(assay type~region + age class + sex). Region, age class, or sex noted in the table indicates a statistically significant relationship with the reference (P-value <0.05). NA indicates no statistically significant relationship.  (+) or (-) indicates the direction of the estimate. (RStudio version 2023.12.1+402).

|  | **Region** | | | | | **Age Class** | | | **Sex** |
| --- | --- | --- | --- | --- | --- | --- | --- | --- | --- |
|  | **DEVA** | **MOJA** | **NW** | **PEBS** | **SNBS** | **2-5 years** | **6+ years** | **young** | **sex** |
| Neutrophils | MOJA (-), PEBS (-), SNBS (+) | DEVA (+), NW (+), PEBS (-), SNBS (+) | MOJA (-), PEBS (-) | DEVA (+), MOJA (+), NW (+), SNBS (+) | DEVA (-), MOJA (-), PEBS (-) | young (-) | young (-) | 2-5 (+), 6+ (+) | NA |
| Lymphocytes | MOJA (+), PEBS (+) | MOJA (-), NW (-), SNBS (-) | MOJA (+), PEBS (+) | DEVA (-), NW (-), SNBS (-) | MOJA (+), PEBS (+) | young (+) | young (+) | 2-5 (-), 6+ (-) | NA |
| Monocytes | NA | PEBS (+) | NA | MOJA (-) | NA | NA | young (-) | 6+ (+) | NA |
| Eosinophils | PEBS (+) | PEBS (+) | PEBS (+) | DEVA (-), MOJA (-), NW (-) , SNBS (-) | PEBS (+) | NA | NA | NA | NA |
| Basophils | NA | NA | NA | NA | NA | 6+ (+) | 2-5 (-) | NA | NA |
| LPA ConA | NA | NA | NA | NA | NA | NA | NA | NA | NA |
| LPA LPS | NA | NA | NA | NA | NA | NA | NA | NA | NA |
| WBBKA | NW(+), PEBS(+) | NW(+), PEBS(+) | DEVA (-), MOJA (-), SNBS (-) | DEVA (-), MOJA (-), SNBS (-) | NW(+), PEBS(+) | 6+ (-) | 2-5 (+) | NA | NA |
| PBKA Max | PEBS (+), SNBS (-) | PEBS (+), SNBS (-) | PEBS (+) | DEVA (-), MOJA (-), NW (-) , SNBS (-) | DEVA (+), MOJA (+), PEBS (+) | young (+) | young (+) | 2-5 (-), 6+ (-) | Male (-) |
| PBKA 50% | MOJA (-) | DEVA (+), NW (+), PEBS (+), SNBS (+) | MOJA (-) | MOJA (-) | MOJA (-) | NA | NA | NA | NA |
| IFNy | MOJA (-), PEBS (-), SNBS (-) | DEVA (+) | NA | DEVA (+) | DEVA (+) | NA | NA | NA | NA |
| IL4 | MOJA (-), NW (+) | DEVA (+), NW (+) | DEVA (-), MOJA (-), PEBS (-) , SNBS (-) | NW (+) | NW (+) | NA | NA | NA | NA |
| IL6 | PEBS (-) | PEBS (-), SNBS (-) | NA | DEVA (+), MOJA (+) | MOJA (+) | NA | NA | NA | NA |
| IL10 | MOJA (-), PEBS (-), SNBS (-) | DEVA (+), NW (+) | MOJA (-), PEBS (-), SNBS (-) | NW (+) | DEVA (+), NW (+) | NA | NA | NA | NA |
| IL17a | MOJA (+), PEBS (-), SNBS (-) | DEVA (-), MOJA (-), PEBS (-) , SNBS (-) | MOJA (+) | DEVA (+), MOJA (+) | DEVA (+), MOJA (+) | NA | NA | NA | NA |
| TNFa | MOJA (-), PEBS (-), SNBS (-) | DEVA (+), NW (+) | MOJA (-), PEBS (-), SNBS (-) | DEVA (+), NW (+) | DEVA (+), NW (+) | NA | NA | NA | NA |
| Albumin | NW(+), SNBS(+) | NW(+), PEBS (+), SNBS(+) | DEVA (-), MOJA (-), PEBS (-) | MOJA (-), NW (+), SNBS(+) | DEVA (-), MOJA (-), PEBS (-) | 6+ (-) | 2-5 (+) | NA | NA |
| Globulins | MOJA (+), PEBS (+) | DEVA (-), NW (-), PEBS (+) , SNBS (-) | MOJA (+), PEBS (+) | DEVA (-), MOJA (-), NW (-) , SNBS (-) | MOJA (+), NW(-), PEBS (+) | 6+ (+), young (-) | 2-5 (-), young (-) | 2-5 (+), 6+ (+) | NA |
| Stimualted PBMC IFNy | MOJA (-), PEBS (-), SNBS (-) | DEVA (+) | NA | DEVA (+) | DEVA (+) | NA | NA | NA | NA |

Table S3: Quantitative results of glms run: Link to qualitative glm raw results table: Figshare, Weinstein 2025; https://doi.org/10.6084/m9.figshare.30680816

Table S3: Intra-plate coefficients of variation for cytokine plates:

| Analyte | Intra Plate Average CV |
| --- | --- |
| IFNy | 18.90 |
| IL-4 | 21.47 |
| IL-6 | 24.62 |
| IL-10 | 12.12 |
| IL-17a | 22.57 |
| TNFa | 11.98 |
