# Supplementary figures and images for: Out of the shed and into the field: an immune toolkit for measuring wild ungulate immune phenotypes at multiple scales
Source: Discov Immunol. 2026 Mar 5;5(1):kyag001. doi: 10.1093/discim/kyag001 (PMC12961423; doi:10.1093/discim/kyag001)

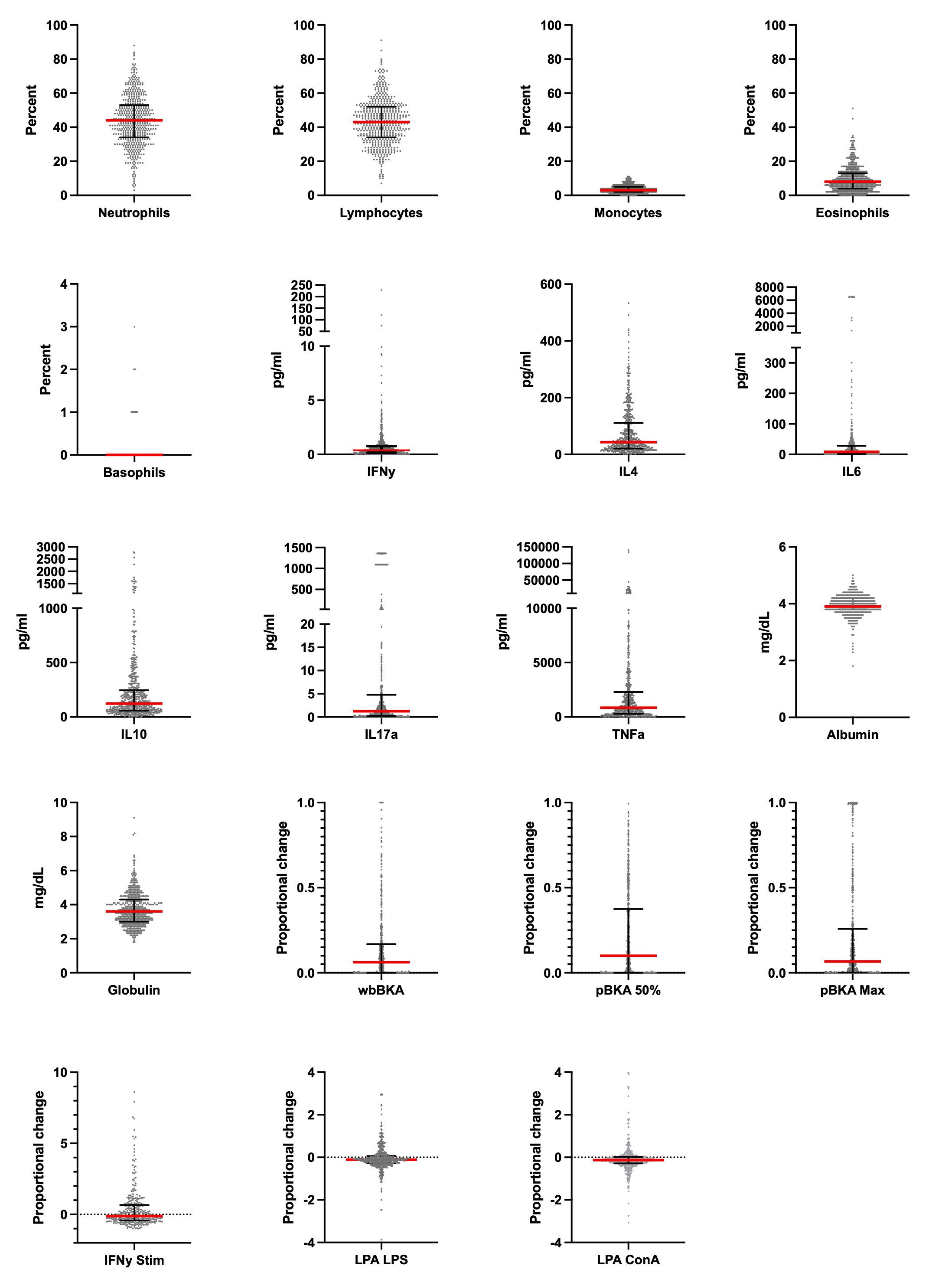

Supplement: kyag001_Supplementary_Data [file kyag001_supplementary_data.zip › Figure S1.tiff]
